# Autosomal dominant optic atrophy caused by six novel pathogenic *OPA1* variants and genotype–phenotype correlation analysis

**DOI:** 10.1186/s12886-022-02546-0

**Published:** 2022-07-26

**Authors:** Jinfeng Han, Ya Li, Ya You, Ke Fan, Bo Lei

**Affiliations:** 1 Department of Ophthalmology, Zhengzhou University People’s Hospital, Henan Provincial People’s Hospital, Zhengzhou University, Zheng-zhou, 450003 China; 2 Academy of Medical Sciences, Zhengzhou University, Zhengzhou, 450000 China; 3 Henan Eye Institute, Henan Eye Hospital, Henan Provincial People’s Hospital, 7 Weiwu Road, Zhengzhou, 450003 Henan China

**Keywords:** ADOA, *OPA1*, Variant, Targeted next-generation sequencing, Chinese, Optic nerve

## Abstract

**Purpose:**

To describe the genetic and clinical features of nineteen patients from eleven unrelated Chinese pedigrees with *OPA1*-related autosomal dominant optic atrophy (ADOA) and define the phenotype-genotype correlations.

**Methods:**

Detailed ophthalmic examinations were performed. Targeted next-generation sequencing (NGS) was conducted in the eleven probands using a custom designed panel PS400. Sanger sequencing and cosegregation were used to verify the identified variants. The pathogenicity of gene variants was evaluated according to American College of Medical Genetics and Genomics (ACMG) guidelines.

**Results:**

Nineteen patients from the eleven unrelated Chinese ADOA pedigrees had impaired vision and optic disc pallor. Optical coherence tomography showed significant thinning of the retinal nerve fiber layer. The visual field showed varying degrees of central or paracentral scotoma. The onset of symptoms occurred between 3 and 24 years of age (median age 6 years). Eleven variants in *OPA1* were identified in the cohort, and nine novel variants were identified. Among the novel variants, two splicing variants c.984 + 1_984 + 2delGT, c.1194 + 2 T > C, two stop-gain variants c.1937C > G, c.2830G > T, and one frameshift variant c.2787_2794del8, were determined to be pathogenic based on ACMG. A novel splicing variant c.1316-10 T > G was determined to be likely pathogenic. In addition, a novel missense c.1283A > C (p.N428T) and two novel splicing variants c.2496G > A and c.1065 + 5G > C were of uncertain significance.

**Conclusions:**

Six novel pathogenic variants were identified. The findings will facilitate genetic counselling by expanding the pathogenic mutation spectrum of *OPA1*.

## Introduction

Autosomal dominant optic atrophy (ADOA, MIM#165,500) is one of the most common hereditary optic neuropathies, with an estimated prevalence ranging from 1:10,000 to 1:30,000 worldwide [1–4]. It is mainly characterized by progressive symmetric painless visual impairment and optic atrophy caused by the degeneration of retinal ganglion cells and their axons in early childhood. More than 20% of the ADOA patients may present with one or more additional features, such as neurosensory hearing loss, progressive external ophthalmoplegia, ptosis, peripheral neuropathy, cataracts or ataxia, syndromic Parkinsonism and dementia[5], which are named ADOA ‘plus’[6]. The penetrance of ADOA varies from 43 to 100% in different families with different mutations [3].

To date, 13 genes, including *OPA1, OPA2, OPA3, OPA4, DNM1L, OPA6, TMEM126A, OPA8, ACO2, RTN4IP1, YME1L1, AFG3L2* [7], *SSBP1* [8] have been found to be associated with hereditary optic atrophy. The *OPA1* gene variants contribute 57 ~ 89% to ADOA [9, 10]. Localized on 3q28-q29, the gene *OPA1* spans more than 100 kb and includes 31 exons, namely exons 1 to 29, exon 4b, and exon 5b. Among them, exon 29 is nonprotein-coding. Alternative splicing of exons 4, 4b and 5b generates eight transcript isoforms [11], which are widely present in tissues, while the expression levels in different tissues vary [12]. The main isoform expressed in the human retina is isoform 1 (NM_015560.2) without 4b and 5b [9], and it was originally identified and frequently used to describe most variants in the *OPA1* gene.

The *OPA1* gene encodes a mitochondrial dynamin-related GTPase family protein [13] of 907 ~ 1015 amino acids, located in the inner mitochondrial membrane and involved in the formation of mitochondrial cristae and mitochondrial fusion. The OPA1 protein contains five domains, three of which are conserved domains: the GTPase domain (exons 8–15), dynamin central region (exons 16–24), and GTPase effector domain (exons 27–28) [14].

Having enabled an efficient and credible detection of gene mutations [15], high-throughput sequencing (HTS) makes the extensive molecular diagnosis possible. Here, we aimed to describe the genetic and clinical features of nineteen patients with identified *OPA1* variants in eleven unrelated Chinese ADOA pedigrees and determine the pathogenicity of these novel variants.

## Methods

### Participants & clinics

This research was conducted in accordance with the tenets of the Declaration of Helsinki and approved by the Ethics Committee of the Henan Eye Hospital. All the participants or their guardians signed informed consents forms (HNEECKY-2019 (15), 15 October 2019). The eleven unrelated Chinese families with optic atrophy were outpatients of the Henan Eye Hospital. Detailed clinical data, including age of onset, disease duration, family history, best-corrected visual acuity (BCVA), fundus photography, optical coherence tomography (SS-OCT, VG200, Henan China), visual field (VF), full-field electroretinogram (ERG) and visual evoked potential (VEP), were collected.

### DNA sample collection & targeted next-generation sequencing

Peripheral blood samples were collected from the participants of the eleven ADOA families and preserved at -20 ℃ before further analysis. Total genomic DNA was extracted with a whole blood DNA extraction kit (TIANGEN, Beijing, China) and quantified with Qubit 4.0.

Targeted next-generation sequencing was performed with a custom designed panel PS400 [16–18], which contains 376 inherited retinal dystrophies and other posterior segment eye disease genes, the 50 bp next to the exons and the known pathogenic/likely pathogenic variants in the introns of the genes. Genomic DNA was randomly sonicated into fragments of approximately 150–200 bp to prepare Illumina paired-end libraries. The DNA fragments were end-repaired, and an extra ‘A’ base was added to the 3' end. Illumina adapters were ligated to the ends of the DNA fragments, and PCR amplification was performed for each sample. PCR amplification was used to enrich the target gene with the specific index and RNA probe. The DNA libraries were quantified by Qubit 4.0. The enriched DNA libraries were sequenced on an Illumina Nextseq500 system (Illumina, San Diego, CA).

### Bioinformatics analysis and sanger sequencing verification

The raw reads were aligned to the human genome reference (USUC hg19) using the Burrows Wheeler Aligner (BWA). Single-nucleotide variants (SNVs) and InDels (Insertions and Deletions) were called by Atlas-SNP2 and Atlas-Indel, respectively. Variant-filtering was based on public and in-house SNP databases, including the Exome Aggregation Consortium database (ExAC), the Genome Aggregation Database (gnomAD, http://gnomad.broadinstitute.org/), Human Genetic Variation Database (HGVD, http://www.genome.med.kyoto-u.ac.jp/SnpDB/), the 1000 Genomes Project database (1000 Genomes, http://browser.1000genomes.org), and the UK10K databases, as well as our internal database, with allele frequency cut-offs of 2% and 0.1% for recessive and dominant variants, respectively. We used three commercial software programs, XYGeneRanger 2.0 (Xunyin, Shanghai, China), TGex (LifeMap Sciences, Alameda, CA, USA) and Efficient Genosome Intepration System, EGIS (SierraVast Bio-Medical Technology Co., Ltd, Shanghai, China), to analyse the pathogenicity of the mutations. The nonsynonymous and splicing variants were analysed by in silico gene function prediction software such as PolyPhen-2 (Polymorphism Phenotyping v2, http://genetics.bwh.harvard.edu/pph2), SIFT (Sorting Intolerant From Tolerant, http://sift.jcvi.org) [19], MutationTaster (http://www.mutationtaster.org) [20], PROVEAN (Protein Variation Effect Analyzer, http://provean.jcvi.org/index.php), and CADD (Combined Annotation Dependent Depletion, https://cadd.gs.washington.edu/) [21]. NetGene2 (http://www.cbs.dtu.dk/services/NetGene2), NNSplice (http://www.fruitfly.org/seq_tools/splice.html) and FSPLICE (http://www.softberry.com/berry.phtml?topic=fsplice&group=programs&subgroup=gfind) were used to predict the splicing defects. Genomic Evolutionary Rate Profiling (GERP)[22] software was applied to identify constrained loci. Project HOPE (http://www.cmbi.umcn.nl/hope) was used to analyze the structural effects of missense variants. The variants were further validated and segregated by Sanger sequencing from all available family members. The pathogenicities of all variants were classified according to the American College of Medical Genetics and Genomics (ACMG)[23] standards and guidelines.

## Results

### Clinical characteristics of the ADOA patients

**Fig. 1:**
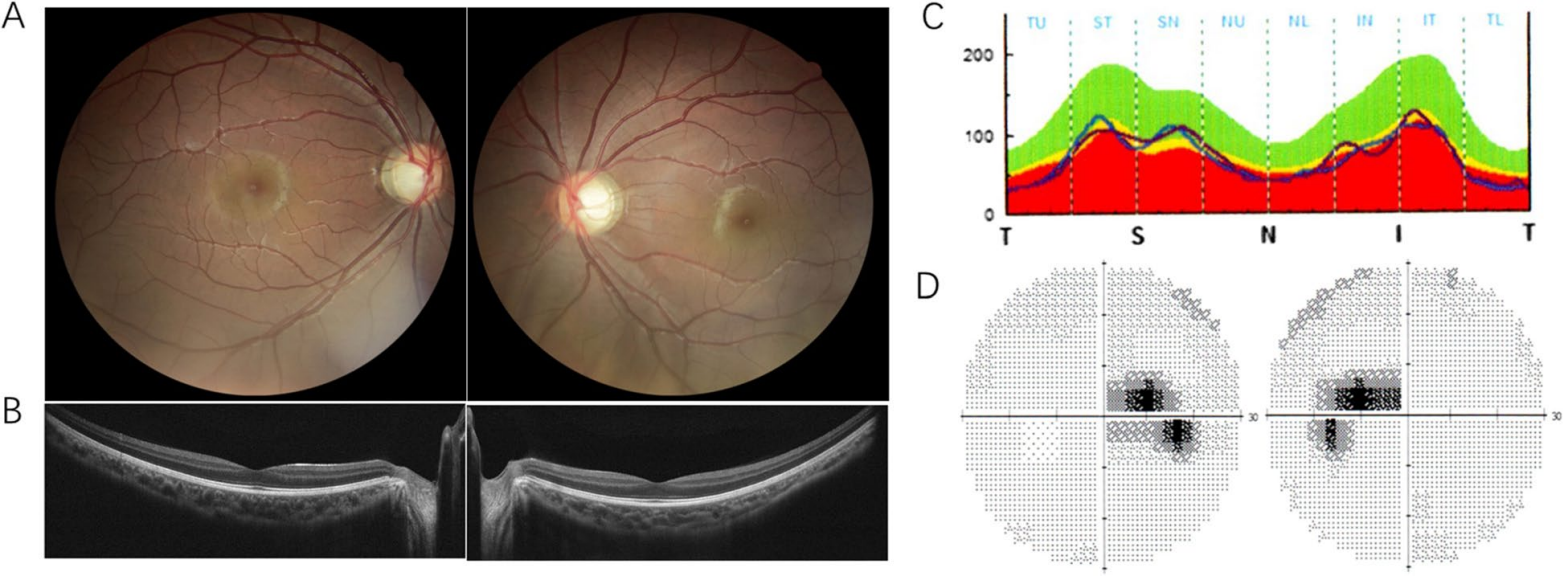
Clinical features of a representative DOA patient, F6-II:2. **A** fundus images, temporal pallor of the ONH; **B** OCT, thinning inner retinal neuroepithelial layer, **C** diffuse thinning of the RNFL. OD, the blue solid line, OS, the purple solid line. **D** central scotoma

There were 33 participants in the 11 pedigrees, and 21 carried variants in *OPA1*. Nineteen of them, including 9 males and 10 females, presented the phenotype. The clinical characteristics of the patients are summarized in Table 1. The age of the patients ranged from 4 to 64 years, with a median age of 12 years. The onset of symptoms occurred between 3 and 24 years of age, and the median was 6 years. Painless progressively symmetric insidious vision loss was the chief complaint in all 19 patients. The disease onset of most cases was from childhood, but the reported age of onset was 24 years for patient F7- III:3. F9-IV:2 showed dyserythrochloropsia. For the most recent evaluation, BCVA varied from finger count to 0.5, and optic atrophy with temporal pallor or diffuse pallor was seen in most cases. Figure 1 shows the clinical features of a representative DOA patient F6-II:2. Two heterozygous mutation carriers, F2-I:1 and F8-I:1, however, showed a normal phenotype with neither vision loss nor optic atrophy. OCT was performed on the father F2 I-1 but not F8 I-1, so a subclinical optic nerve atrophy can’t be excluded for F8 I-1. OCT showed significant thinning of the retinal nerve fiber layer (RNFL). VF showed varying degrees of predominantly central or paracentral scotoma, even temporal hemianopsia, for patient F11-II:2, who presented with external ophthalmoplegia in addition to optic atrophy. No other extraocular neurologic features were observed.

**Table 1:** Clinical characteristics of the ADOA patients in the study

Patient	Sex	Onset Age (y)	Age at Diagnosis (y)	BCVA (OD/OS)	ONH	RNFL (OD/OS)	VF	VEP	Brain MRI
F1-I:1	M	NA	64	0.05/0.01	TP	TT	CS	NA	NA
F1-II:3	F	NA	34	0.4/0.2	TP	TT	CS	NA	NA
F1-III:3	M	5	5	0.5/0.5	DP	DT	NA	NA	N
F2-I:1	M	NA	42	1.0/1.0	N	N	NA	NA	NA
F2-II:2	F	7	7	0.2/0.2	DP	DT	CS	PL	N
F3-II:2	M	NA	33	0.25/0.3	TP	TT	NA	NA	NA
F3-III:3	M	6	6	0.25/0.25	TP	TT	NA	NA	NA
F4-III:1	F	10	10	0.3/0.25	TP	NA	NA	NA	NA
F5-III:1	M	NA	35	0.5/0.5	TP	NA	NA	NA	NA
F5-IV:1	M	8	11	0.3/0.2	TP	TT	CS, PS	PL, DA	NA
F6-II:2	F	7	12	0.2/0.2	TP	TT	CS	DA	N
F7-III:3	M	24	24	0.2/0.25	TP	TT	S↓	DA	NA
F8-I:1	M	NA	36	1.0/1.0	N	NA	NA	NA	NA
F8-II:1	M	5	7	0.1/0.1	TP	NA	S↓	NA	N
F9-III:3	F	3	33	0.12/0.12	TP	TT	NA	NA	NA
F9-IV:2	F	5	6	0.25/0.15	TP	TT	NA	PL, DA	NA
F10-III:1	F	12	35	0.4/0.4	TP	TT	NA	NA	NA
F10-III:3	F	NA	29	0.3/0.4	TP	TT	NA	NA	NA
F10-IV:1	M	5	5	0.5/0.4	TP	TT	NA	NA	N
F11-II:2	F	3	29	0.01/FC	TP	TT	TH	PL, DA	N
F11-III:1	F	3	4	NA	TP	TT	NA	NA	NA

*BCVA* best corrected visual acuity, *ONH* optic nerve head, *RNFL* retinal nerve fiber layer, *VF* visual field, *VEP* visual evoked potential, *MRI* magnetic resonance imaging, *M* male, *F* female, *NA* not available, *TP* temporal pallor, *DP* diffuse pallor, *DT* diffuse thinning, *TT* temporal thinning, *CS* central scotoma, *PS* paracentral scotoma, *S* sensitivity, *TH* temporal hemianopsia, *PL* prolonged latencies, *DA* diminished amplitudes, *N* normal

### Genetic analysis of the ADOA patients

**Fig. 2:**
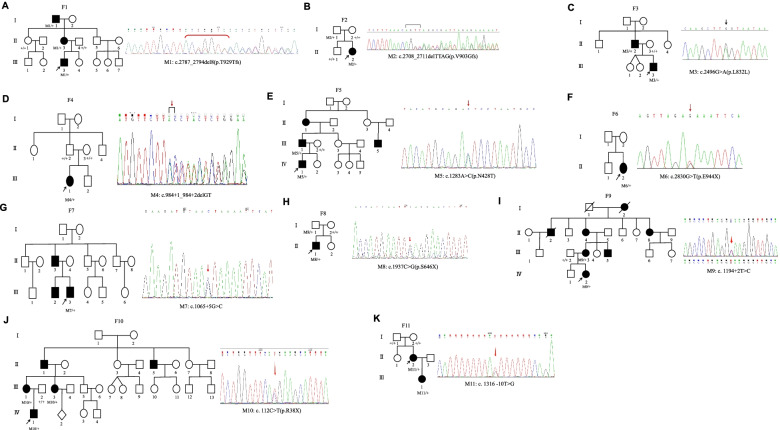
Pedigrees and sequencing results of the 11 *OPA1*-related ADOA families. **A** F1-I:1, F1-II:3 and F1-III:3 carried the heterozygous c.2787_2794del8 variant. **B** F2-I:1 and F2-II:2 carried the heterozygous c.2708_2711delTTAG variant. **C** F3-II:2 and F3-III:3 carried the heterozygous c.2496G > A variant. **D** F4-III:1 carried the de novo heterozygous c.984 + 1_984 + 2delGT variant. **E** F5-III:1 and F5-IV:1 carried the heterozygous c.1283A > C (p.N428T) variant. **F** F6-II:2 carried the heterozygous c.2830G > T variant. **G** F7-III:3 carried the heterozygous c.1065 + 5G > C variant. **H** F8-I:1 and F8-II:1 carried the heterozygous c.1937C > G variant. **I** F9-III:3 and F9-IV:2 carried the heterozygous c.1194 + 2 T > C variant. **J** F10-III:1, F10-III:3 and F10-IV:1 carried the heterozygous c.112C > T variant. **K** F11-II:2 and F11-III:1 carried the heterozygous c.1316-10 T > G variant

We performed targeted next-generation sequencing (NGS) in the 11 ADOA families, and identified five splicing variants, c.2496G > A, c.984 + 1_984 + 2delGT, c.1065 + 5G > C, c.1194 + 2 T > C and c.1316-10 T > G, two frameshift deletion variants, c.2708_2711delTTAG and c.2787_2794del8, three stop-gain variants, c.2830G > T, c.1937C > G and c.112C > T, and a missense variant, c.1283A > C (p.N428T) in the *OPA1* gene. Figure 2 shows the pedigrees and sequencing results of the 11 ADOA families. The disease showed autosomal-dominant inheritance, and 7 of the variants were significantly cosegregation with the disease. F2-II:2 and her father carried the same heterozygous c.2708_2711delTTAG frameshift variant. F8-II:1 and his father shared the same c.1937 C > G (p.S646X) stop-gain variant. However, F2-II:2 and F8-II:1 showed typical optic nerve atrophy, while their fathers had a normal phenotype. F4-III:1 carried a de novo heterozygous splicing variant c.984 + 1_984 + 2delGT. F11-II:2 carried a de novo heterozygous splicing variant c.1316-10 T > G. Neither of their parents had the same variants, but neither of their paternities was checked.

**Fig. 3:**
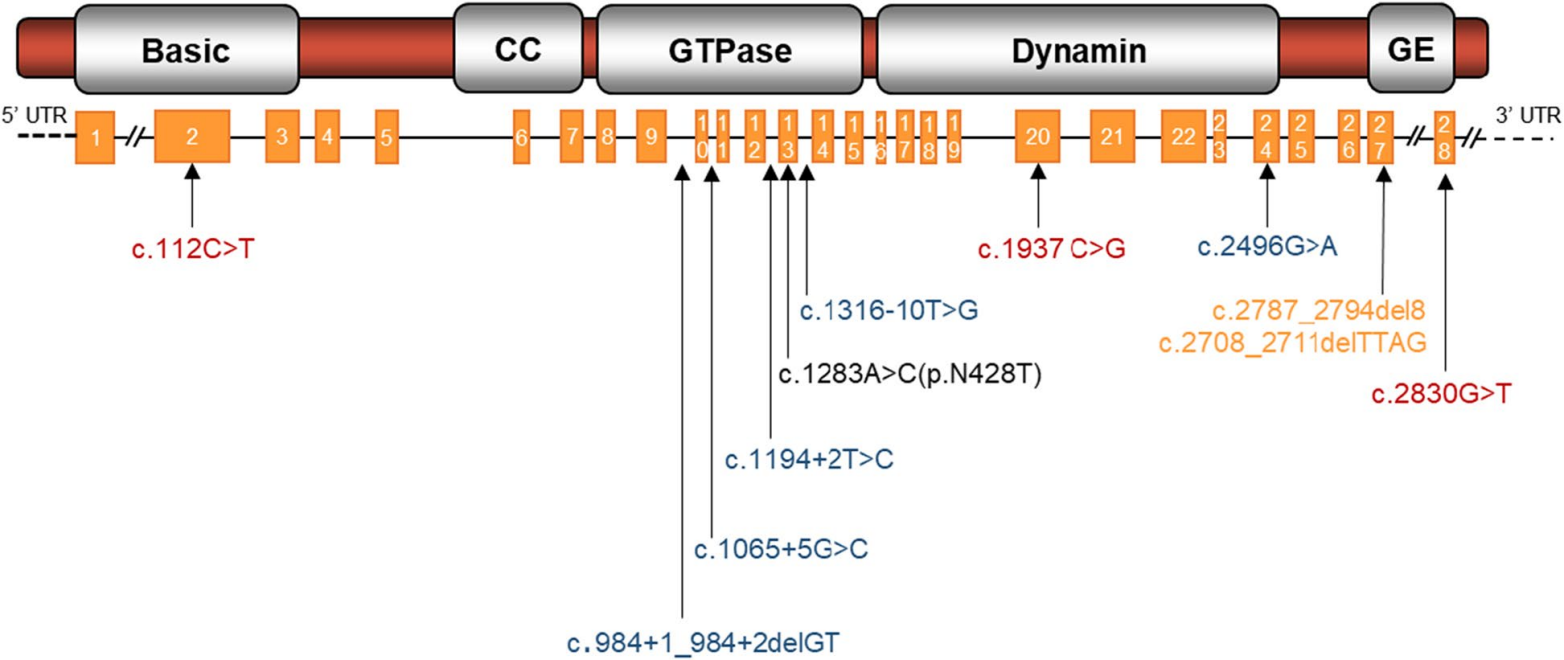
All 11 *OPA1* mutations identified in this study are shown in the schematic diagram of the *OPA1* gene (Ref. NM_015560.2, below) and OPA1 protein (above). Missense, splicing, stop-gain and frameshift deletion mutations are coloured in black, blue, red and yellow, respectively. CC, coiled coil domain; GE, GTPase effector domain

All 11 *OPA1* mutations identified are demonstrated in the schematic diagram of the *OPA1* gene (Ref. NM_015560.2) and protein (Fig. 3). Among these variants, five (5/11, 45.5%) are located at the GTPase domain, three (3/11, 27.3%) at the GTPase effector (GE) domain, two (2/11, 18.2%) at the dynamin central region domain and one (1/11, 9.1%) at the basic domain. In addition, six of these mutations occurred in the exons, while the other occurred in introns.

Pathogenicity analyses of *OPA1* variants in the 11 Chinese patients are summarized in the Table 2. According to ACMG standards and guidelines, of the 11 *OPA1* variants, nine novel undocumented variants were identified. Five novel variants were classified as pathogenic: two splicing variants, c.984 + 1_984 + 2delGT and c.1194 + 2 T > C, two stop-gain variants, c.1937C > G and c.2830G > T, and one frameshift variant, c.2787_2794del8. A novel splicing variant c.1316-10 T > G was determined to be likely pathogenic. Among the above, the two variants c.984 + 1_984 + 2delGT and c.1316-10 T > G were de novo. A novel missense variant, c.1283A > C (p.N428T), and two novel splicing variants, c.2496G > A and c.1065 + 5G > C, were of uncertain significance. In addition, the pathogenic stop-gain variant c.112C > T (OPA1_000236, Variant #0000760813 (NC_000003.11: g.193332591C > T, OPA1(NM_015560.2):c.112C > T)—Global Variome shared LOVD) has been reported in ADOA families [24].

**Table 2:** *OPA*1 variants identified from 11 unrelated Chinese ADOA families and pathogenicity analyses

family ID	Position	Nucleotide change		Protein variant	Mutation type	SIFT	MutationTaster	PROVEAN	Net Gene2	CADD	gnomAD	ExAc	1000 Genomes	ACMG	Evidence	Novel
		NM_015560.2	NM_130837.2													
F1	Exon 27	c.2787_2794del	c.2952_2959del	p.T929Tfs	frameshift deletion	-	DC(1)	-	-	-	-	-	-	P	PVS1 + PM2 + PP1	Y
F2	Exon 27	c.2708_2711del	c.2873_2876del	p.V903Gfs	frameshift deletion	-	DC(1)	-	-	-	3.658E-5	3.369E-5	-	P	PVS1 + PS4 + PM1 + PM2	N
F3	Exon 24	c.2496G > A	c.2661G > A	p.L832L	splicing	-	DC(1)	-	SSC	D(28)	-	-	-	VUS	PM2 + PP3 + PP4	Y
F4	Intron 9	c.984 + 1_984 + 2del	c.1149 + 1_1149 + 2del	-	splicing	-	DC(1)	-	SSC	-	-	-	-	P	PVS1 + PM6 + PM2	Y
F5	Exon 13	c.1283A > C	c.1448A > C	p.N428T	missense	D(0.003)	DC(1)	D(-5.74)	-	D(27.7)	-	-	-	VUS	PM2 + PP3 + PP4	Y
F6	Exon 28	c.2830G > T	c.2995G > T	p.E944X	stop-gain	-	DC(1)	-	-	D(54)	-	-	-	P	PVS1 + PM2 + PP4	Y
F7	Intron 10	c.1065 + 5G > C	c.1230 + 5G > C	-	splicing	-	DC(1)	-	SSC	D(26.3)	-	-	-	VUS	PM2 + PP3 + PP4	Y
F8	Exon 20	c.1937 C > G	c.2102C > G	p.S646X	stop-gain	-	DC(1)	-	-	D(41)	-	-	-	P	PVS1 + PM2 + PP4	Y
F9	Intron 12	c.1194 + 2 T > C	c.1305 + 2 T > C	-	splicing	-	DC(1)	-	SSC	D(32)	-	-	-	P	PVS1 + PM2 + PP4	Y
F10	Exon 2	c.112C > T	c.112C > T	p.R38X	stop-gain	-	DC(1)	-	-	D(38)	-	-	-	P	PVS1 + PM2 + PP1 + PP4	N
F11	Intron 13	C.1316-10 T > G	c.1478-10 T > G	-	splicing	-	DC(1)	-	SSC	D(29)	-	-	-	LP	PM6 + PM2 + + PP3 + PP4	Y

*D* deleterious (<0.05), *DC* disease causing, *SSC* splicing site change, *P* pathogenic, *LP*, likely pathogenic, VUS variant of uncertain significance, − not available

## Discussion

ADOA represents autosomal dominant optic atrophy, and 57 ~ 89% of ADOA cases are caused by variants in the *OPA1* gene. According to the Leiden Open Variation Database (LOVD, https://www.lovd.nl/), the original eOPA1 database, more than 400 *OPA1* pathogenic variants have been reported (https://databases.lovd.nl/shared/genes/OPA1). Among them, 28% are missense variants, 24% are associated with altered splicing, 22% are frameshift variants, 15% are nonsense variations, and 7% are deletions [25]. In this study, 11 probands from 11 unrelated Chinese ADOA families presented varying vision defects and optic disc pallor, and were all identified as carrying heterozygous *OPA1* variants. Not consistent with LOVD, among these variants, splicing variants (5/11, 45.5%) were the most common mutation type, followed by the stop-gain (3/11, 27.3%), frameshift deletion (2/11, 18.2%), and missense variants (1/11, 9.1%), which were the most common in the LOVD *OPA1* database. The difference may be related to race or the smaller sample size in this study. On the other hand, missense mutations are more likely to develop ADOA ‘plus’ phenotypes [26, 27], while the only missense variant in our study did not present with plus phenotypes other than ocular signs.

The penetrance of ADOA varies from 43 to 100% in different families with different mutations [3]. We also observed the incomplete penetrance of ADOA. The heterozygous mutation carriers F2-I:1 (c.2708_2711delTTAG) and F8-I:1 (c.1937C > G) had a normal phenotype with neither decreased visual acuity nor optic atrophy. Therefore, for those who have atypical phenotypes, genetic tests are essential to make a correct diagnosis.

Haploinsufficiency and dominant negative effects contribute to the pathogenesis of the *OPA1*-related ADOA [28]. Haploinsufficiency indicates that pathogenic *OPA1* variants lead to impaired OPA1 functions by reducing the expression of OPA1 protein. In our study, regardless of where the variant loci and protein domains were located, all splicing, stop-gain and frameshift deletion variants, were predicted to cause reduced OPA1 protein. Haploinsufficiency should be their pathogenesis. In addition, the variant types seemed to have no correlation with the severity of vision defects, which was in agreement with Xu et al. [29].

On the other hand, previous studies have shown that some missense mutations in the GTPase domain of OPA1 do not reduce the expression of OPA1. The mutated protein may compete with the wild-type protein and inhibit the function of OPA1, resulting in a dominant negative effect, thereby interfering with OPA1 functions. F5-IV:1, a 10-year-old boy, and his father F5-III:1 both carried the heterozygous c.1283A > C (p.N428T) missense variant and presented with vision impairment and optic atrophy. Just as most missense pathogenic variants reported for the *OPA1* gene were clustered in the highly conserved GTPase domain, the only missense variant c.1283A > C (p.N428T) in the current study was also located in exon 13 in the GTPase domain, which may be caused by a dominant negative effect.

Clearly, the documentation of dominant negative effect diseases is of great significance. It has been confirmed in animal studies and clinical trials that autosomal recessive inherited diseases could benefit from gene augmentation therapy. However, gene therapy of autosomal dominant diseases remains a challenge. To date, three *Opa1* mouse models carrying the truncation mutations (c.1051C > T, c.1065 + 5G > A, c.2708-2711delTTAG) [30–33] and showing haploinsufficiency have been tested for gene therapy. Unlike haploinsufficiency, simple augmentation of normal OPA1 levels may not be effective for gene therapy of missense mutations because of a dominant-negative mode of action [34]. Therefore, it is necessary to develop mouse models carrying missense mutations in *OPA1* and presenting dominant negative effects. In addition, missense mutations tend to develop ADOA “plus” [27], which also impairs multiple systems such as the musculoskeletal system, nervous system and circulation system. It is also a challenge to develop therapeutic strategies for diseases affecting multiple organs.

The GTPase domain, dynamin central region, and GTPase effector domain are conserved [14]. More than 50% of the pathogenic *OPA1* variants are located in the GTPase domain and the GTPase effector domain (exons 27–28) [35]. Similar to the database, among the variants identified in this study, which mainly affected the coding sequence and exon–intron boundaries of the gene (six of these mutations occurred at the exons, while the other occurred at the introns), eight (8/11, 72.7%) were located in the two domains, highlighting the importance of these domains in OPA1 protein functions. Impairment of GTPase activity could decrease the stability of the inner mitochondrial membrane structure and membrane potential because of proton leakage [36]. Additionally, according to a meta-analysis of genotype–phenotype analysis of *OPA1*-related ADOA, the most common exon involved was exon 27 [37]. Two frameshift variants, c.2787_2794del8 and c.2708_2711delTTAG, were in exon 27, which might lead to the premature termination of OPA1 protein synthesis and truncated proteins and protein degradation or nonsense-mediated mRNA decay. It is worth mentioning that the hotspot variant *OPA1* gene c.2708_2711delTTAG identified in this study has been reported multiple times and could account for approximately 10% of ADOA [9, 38, 39]. In addition, variants in the CC domain were rarely reported in the LOVD database, and there was no variant identified in the CC domain in this study due to the small sample size.

According to ACMG standards and guidelines, we defined eight pathogenic variants (one variant was classified as likely pathogenic) of the 11 *OPA1* variants. Three novel variants, including a missense variant c.1283A > C (p.N428T), and two splicing variants, c.2496G > A and c.1065 + 5G > C, were of uncertain significance. The HOPE online software revealed that the missense variant c.1283A > C (p.N428T) could change the physico-chemical parameters or structure of the OPA1 protein. Alavi et al. reported a mutation c.1065 + 5G > A, which is in the same location as c.1065 + 5G > C. They confirmed in a mouse model carrying c.1065 + 5G > A in the *Opa1* gene that c.1065 + 5G > A induced a skipping of exon 10 during transcript processing and led to an in-frame deletion of 27 amino acid residues in the GTPase domain [30]. Multiple software programs (NetGene2, NNSplice and FSPLICE) predicted that the splicing variant c.2496G > A changed the donor splice sites.

In conclusion, we identified nine novel and two reported variants of the *OPA1* gene from 11 unrelated Chinese ADOA families. All 19 patients had varying impaired vision and signs. In addition, we defined six novel pathogenic variants of the 11 *OPA1* variants. Medical genetic tests are essential to make a diagnosis for those who have atypical phenotypes because of incomplete penetrance. An integrated comprehension of the clinical and genetic spectrum of ADOA would certainly advance therapeutic approaches.

## Data Availability

Data presented in this study are contained within the article.
